# Nutritional Disparities among Women in Urban India

**DOI:** 10.3329/jhpn.v31i4.20052

**Published:** 2013-12

**Authors:** Siddharth Agarwal, Vani Sethi

**Affiliations:** Urban Health Resource Centre, New Delhi 110 029, India

**Keywords:** Sl Anthropometry, Equation, Gestational age, New Ballard Score, Newborn, India

## Abstract

The paper presents a wealth quartile analysis of the urban subset of the third round of Demographic Health Survey of India to unmask intra-urban nutrition disparities in women. Maternal thinness and moderate/severe anaemia among women of the poorest urban quartile was 38.5% and 20% respectively and 1.5-1.8 times higher than the rest of urban population. Receipt of pre- and postnatal nutrition and health education and compliance to iron folic acid tablets during pregnancy was low across all quartiles. One-fourth (24.5%) of households in the lowest urban quartile consumed salt with no iodine content, which was 2.8 times higher than rest of the urban population (8.7%). The study highlights the need to use poor-specific urban data for planning and suggests (i) routine field assessment of maternal nutritional status in outreach programmes, (ii) improving access to food subsidies, subsidized adequately-iodized salt and food supplementation programmes, (iii) identifying alternative iron supplementation methods, and (iv) institutionalizing counselling days.

## INTRODUCTION

India is home to 1.21 billion people (1). Out of them, 377.1 million people (31.2%) live in urban areas (1). India is urbanizing rapidly. Its urban population has increased from 27.8% in 2001 to 31.2% in 2011 and is expected to increase further to 535 million (38%) by 2026 (2).

Cities are recognized as hubs for economic development. However, the din and tumult of urban prosperity mutes the co-existence of hunger, undernutrition and deprivation among urban poor population. According to the Planning Commission of India, which undertakes official estimates of poverty, 25.7% (96.91 million) of India's urban population is ‘poor’, i.e. living below the official national level of poverty line (3). A per-capita expenditure of rupees 578.8 per month in urban areas (at 2004-2005 prices) is considered by the Planning Commission of India as the official poverty line. Urban poor habitations—slums and similar underserved settlements—are characterized by overcrowding, environmental pollution, and lack of or inadequate access to basic water, sanitation, health and nutrition services. Populations living in non-notified slums are even more vulnerable as these settlements are not recognized by authorities and, hence, usually remain outside the purview of health, nutrition, food subsidy, and other civic services (4).

Food inflation, meagre wages, cash-based economy, and involvement in strenuous manual work in informal/unorganized sector, unprotected by labour laws of maternity leave, heighten the risk of undernutrition among urban underprivileged women, thus adversely affecting their future and that of their children.

National and state-level information about maternal nutrition situation among urban poor in India, based on representative datasets, is still not available. This study is among the first attempts to analyze large-scale secondary data, such as from Demographic and Health Survey (DHS) to unmask the extent of disparities in nutritional status and access to services of Indian women belonging to the poorest wealth quartile of the country's urban population compared to the rest of the urban population.

## MATERIALS AND METHODS

The study is based on an analysis of the DHS-3 (2005-2006) data for India. The survey is called the National Family Health Survey (NFHS). This survey collected information on maternal and child health and nutrition for all livebirths in the five years preceding the survey from a sample of 1,24,385 women in the age-group of 15-49 years (pregnant, lactating and non-pregnant, and non-lactating) from 29 Indian states (5). This survey followed stratified sampling technique and used a wealth index. The wealth index is a composite measure of the cumulative living standard of a household used in NFHS-3. It is calculated by combining data on a household's ownership of 33 assets into an index, using the factor analysis procedure. The NFHS-3 sampling methodology and wealth index have been detailed elsewhere (6).

In this study, the urban sample was first segregated from the Indian data to arrive at nutrition indicators for urban poor women. The cutoffs based on wealth index were calculated for the urban sample. The urban sample was divided into quartiles as per wealth index and the lowest quartile taken as representative of the urban poor in India in view of the estimates of the Planning Commission of India, designating 25.7% of the total urban population to be below the poverty line (3). Of the NFHS's sample of urban index population, 22.6% was categorized as poor (lowest urban quartile) by this method. The unweighted number (N) of population in the urban sample included in the analysis was 14,527 while the weighted N was 10,626. On the basis of the wealth index, a total of 3,839 women belonged to the poorest urban quartile category (weighted N 3,095), followed by 3,726 women in the second-lowest urban quartile (weighted N 2,691), 3,601 in the rich category (weighted N 2,598), and 3,361 in the richest urban quartile (weighted N 2,242). The lowest urban quartile was considered ‘urban poor’, and the other three wealth index quartiles were combined and considered ‘rest of urban population’. The analysis was carried out in SPSS (version 12.0) on weighted cases as per the national standard weights applied to account for factors, such as sample probability, non-response, and sampling differences, across regions.

In this study, the following nutrition indicators of the NFHS were analyzed for women of reproductive age (15-49 years): (i) height below 145 cm, also called short stature of women, indicating that a woman is nutritionally at risk; (ii) body mass index <18.5 kg/m^2^, also described as maternal thinness, indicating that the woman is underweight, hence, undernourished; (iii) those who were suffering from mild, moderate and severe anaemia, based on haemoglobin level assessed through cyanmethemoglobin method; (iv) those who were pregnant and reported night blindness, i.e. difficulty in seeing at dusk; and (v) those belonging to households consuming salt with nil iodine content, i.e. 0 parts per million (ppm), inadequate (>0-<15 ppm), and adequate (15 or more ppm) iodine contents. Mild, moderate, severe, and any anaemia were defined as: mild anaemia (haemoglobin level between 10.0 and 10.9 g/dL for pregnant women; haemoglobin level between 10.0 and 11.9 g/dL for non-pregnant women), moderate anaemia (haemoglobin level between 7.0 and 9.9 g/dL), severe anaemia (haemoglobin level <7.0 g/dL), any anaemia (haemoglobin level <11 g/dL for pregnant women; haemoglobin level 12 g/dL for non-pregnant women).

Additionally, the following indicators relating to care-seeking behaviour and access to services were also studied: (i) frequency of consumption of milk and pulses or beans in the past 30 days (weekly, occasionally, and never); (ii) number of days for which iron folic acid tablets were taken during pregnancy; (iii) receipt of a drug for intestinal parasites during pregnancy; and (iv) receipt of supplementary nutrition and nutrition and health education during pregnancy and lactation from an *Anganwadi* Centre. An *Anganwadi* Centre delivers at the grassroots level the services of the largest nutrition programme in India—the Integrated Child Development Services (ICDS).

Sociodemographic profile of the study sample is presented in Table 1.

## RESULTS

### Maternal short stature and thinness

Women who had short stature were 1.7 times higher in the poorest urban quartile compared to rest of the urban population (14.5% vs 8.4%) and 1.5 times higher in poorest urban quartile compared to the urban aggregate (14.5% vs 9.8%) (Table 2). Maternal thinness was 38.5% in the poorest urban quartile, 1.8 times higher compared to the rest of the urban population (21%), and 1.5 times higher than urban aggregate (25%).

### Micronutrient deficiencies

As revealed in Table 2, mild anaemia did not vary markedly across quartiles (36-29%) and affected nearly one-third of urban population in all segments. However, proportion of women belonging to the poorest urban quartile with moderate/severe anaemia (20%) was 1.4 times higher compared to the rest of the urban population (13.7%). Prevalence of moderate/severe anaemia in pregnancy and lactation in the poorest urban quartile was also 1.3 times higher compared to the rest of the urban population.

**Table 1.** Background characteristics of the study sample (%)

Key indicator from NFHS-3	Poorest urban quartile (n=3,095)	Rest of urban population (n=7,531)	Urban aggregate (n=10,626)
Religion			
Hindu	72.4	74.9	75.5
Muslim	24.4	18.7	17.8
Other religious groups	3.2	6.4	6.7
Caste			
Schedule caste	26.8	14.4	17.4
Schedule tribe	5.2	2.7	2.9
Other backward classes	44.2	35.8	37.9
General caste/Others	23.8	47.1	41.8
Educational attainment			
No education/Incomplete primary	64.8	17.3	22.0
Primary	7.9	5.9	12.2
Middle	25.4	44.0	49.4
Secondary and higher	1.9	32.8	16.4
Employment of woman (in the last 12 months)			
No employment	71.0	83.1	79.5
Skilled/Manual labour	11.8	6.0	7.6
Professional/Technical/Managerial	0.2	4.7	3.4
Agricultural employee	8.0	0.8	2.8
Sales worker	1.8	1.4	1.9
Service-holder	6.9	2.9	4.1
Clerical job	0.3	1.1	0.7
Autonomy			
Has no money of her own to use	57.7	47.0	47.9
Has money of her own to use	42.3	53.0	52.1
Ever experienced physical violence from husband	47.2	23.2	29.1
Women with higher (3+) birth orders	28.6	11.4	16.3
Access to sanitary facility at home for disposal of excreta	47.2	95.9	83.2

Night blindness or difficulty in seeing at dusk, a result of chronic vitamin A deficiency, was reported by 7% women in the poorest urban quartile, which was 2.9 times higher than the rest of the urban population (2.4%).

Nearly one-fourth (24.5%) of households in the poorest urban quartile consumed salt with no iodine content, which was 2.8 times higher compared to the rest of the urban population (8.7%).

## DISCUSSION

Wide disparities in maternal undernutrition within urban India across wealth quartiles have been unmasked through this study. This highlights the need for disaggregating urban aggregate data to make nutrition inequities in urban India visible and aligning programmes to address the nutrition needs of this urban disadvantaged segment of the population.

Maternal short stature and thinness indicated by height below 145 cm and low body mass index (less than 18.5 kg/m^2^) respectively, in addition to reduced physical and functional capacity, can negatively affect pregnancy outcomes. It is known that women with height below 145 cm are at a higher risk of a caesarian delivery and delivering a baby with intra-uterine growth restriction (7). Unlike children, nutrition assessment in women, especially pregnancy weight monitoring, followed by corrective action, is not routinely done at field level. Given the large proportion of undernourished urban poor women, there is a need to assess undernutrition among women either periodically by grassroots-level workers in the nutrition and health programmes as part of the Government or NGO outreach programmes, e.g. health and nutrition days, or by students from social work, public health nutrition institutions or medical schools as part of their practicum. Corrective/mitigation measures for women identified as undernourished need to be instituted. These could include providing an additional food supplement to such women (on similar lines as being done for severely-underweight children), enrolling undernourished women for behaviour promotion plus self-confidence enhancement sessions, improving their access to food subsidy and linkage to poverty alleviation programmes since such women often come from food-insecure families owing to irregular/low wages and poor access to food subsidy.

**Table 2.** Nutrition of women in urban India: comparing the poorest urban quartile with rest of the urban population and urban aggregate (%)

Key indicator from NFHS-3	Poorest urban quartile (N=3,095)	Rest of urban population (N=7,531)	Urban aggregate (N=10,626)
Short stature (Height <145 cm)			
Pregnant women	13.9	9.1	10.6
Lactating women	15.1	8.4	10.7
All women	14.5	8.4	9.8
Underweight (BMI <18.5 kg/m2)*			
All women	38.5	21.0	25.0
Mild anaemia			
Pregnant women	25.2	27.7	25.5
Lactating women	46.7	39.3	41.9
All women	38.9	34.9	35.8
Moderate anaemia			
Pregnant women	36.0	24.2	28.0
Lactating women	18.5	13.3	15.1
All women	17.6	12.4	13.6
Severe anaemia			
Pregnant women	2.7	1.3	1.7
Lactating women	2.0	1.2	1.5
All women	2.4	1.3	1.5
Women who took iron-folic acid for 90 days or more while pregnant	18.5	41.8	34.8
Women who took a drug for intestinal parasite while pregnant	3.0	4.9	4.4
Women who reported night blindness during pregnancy	7.0	2.4	3.7
Women who consumed milk/curd on daily basis	29.1	52.8	47.4
Women who consume pulses/beans on daily basis	48.2	63.7	60.1
Women from households consuming adequately-iodized salt (15 ppm or more)	48.1	79.7	71.5
Women from households consuming inadequately- iodized salt (>0-<15 ppm)	27.4	11.6	15.7
Women from households consuming iodized salt with nil iodine content (0 ppm)	24.5	8.7	12.8
Women who reported receiving nutrition and health education from the *Anganwadi* worker			
During pregnancy	15.6	6.0	9.3
During lactation	12.2	4.1	6.7

*Not done in pregnant women and those with infants aged <2 months; Values mentioned in table indicate percentages

Government of India's flagship nutrition programme—the Integrated Child Development Services Scheme—mandates nutrition education (along with other services) to pregnant and lactating women through its frontline workers. The analysis shows that the proportion of women who reported receiving nutrition and health education from an *Anganwadi* worker during their pregnancy and lactation is very low (15.6% for pregnant women and 14.2% for lactating women belonging to the poorest urban quartile). While ICDS coverage and food distribution are monitored as part of programme monitoring and counselling (for behaviour promotion) as an activity in terms of regularity, coverage and quality is often not routinely monitored. Some Indian states, such as Madhya Pradesh, Assam, Rajasthan, and Gujarat, have institutionalized monthly group counselling days at *Anganwadi* centres by frontline workers, using an approach involving fixed day, time, and venue, for pregnant and lactating women to improve regularity, quality, reporting and monitoring of nutrition and health counselling. Such approaches may be adapted and their quality should be improved incrementally.

Food consumption by the poor, in any case, is quite low. Further, when food inflation is high, coupled with pressing non-food expenses, the situation further decreases the proportion of income spent on food (8). In the present study, only 29% women in the poorest wealth quartile consumed milk or curd (a good source of protein) on daily basis. With the rapid food inflation (particularly in the recent six years after this survey), the proportion of urban poor consuming milk/curd can be expected to be much less than this figure. Experiences of food insecurity and hunger among slum households have been reported in other studies (9,10). It is important to improve access to food subsidy in food-insecure settings by addressing gaps in Public Distribution System which offers subsidized access to food and essential commodities, viz. wheat, rice, sugar, and kerosene or in alternative approaches that are being currently debated (11) or piloted (12).

Maternal anaemia during pregnancy increases the risk of perinatal and maternal mortality and contributes to low birthweight (7). While anaemia among pregnant women (Hb <11 g/dL) in the poorest urban wealth quartile, according to the present study, is 64%, other slum-based studies that assessed anaemia in pregnant women, using similar method and cutoff in India, provide figures of 81-93% (13-15). One of the prophylactic measures for anaemia prevention is consumption of one iron folic acid (IFA) tablet every day for a minimum of 100 continuous days during pregnancy, beginning from the second trimester and till 3 months postpartum. If the woman is identified as being moderately to severely anaemic, two tablets each day are recommended. It is disturbing to note that only 18.5% of women in the poorest wealth quartile took IFA tablets for 90 days or more during pregnancy. Intestinal parasitic infections aggravate the existing anaemia (16), and as low as 3% women in the poorest urban wealth quartile received an antihelminthic dose during pregnancy. Abysmally low IFA compliance, coupled with high dietary inadequacy, highlights the need to: (a) regularly counsel mothers to help them overcome difficulty in consuming iron folic acid tablets; (b) in place of ferrous sulphate which is the usual present formulation of the tablets, the use of ferrous fumarate, which has fewer side-effects, should be considered; (c) ensure improved access to cereals and promote consumption of iron-rich vegetables (e.g. different types of green beans) which continue to be the largest source of iron; (d) experiment with strategies to improve compliance to consumption of iron folic acid tablets; (e) test alternative models to reduce iron-deficiency anaemia in such settings. In other developing countries, the use of energy-dense micronutrients—ready-to-use food supplements—has proven successful in the prevention of anaemia and improving birth outcomes (17-18); (f) measures to prevent helminthic infestations would also require to be strengthened, more so in slum settings where many women work or walk barefoot (and hence at a risk of hookworm infestation); and (g) contamination of water is also high, increasing the risk of intestinal worm infestation and calling for expeditious efforts to improve access of the urban poor and deprived populations to safe drinking-water. The risk of hookworm and intestinal worms makes it imperative that national deworming guidelines for pregnant women be established explicitly, mentioning the dosage and frequency of deworming tablets. These should be distributed also in districts where prevalence data for worm infestation are not available at a given point in time.

The present study showed that proportion of women who reported night blindness [a clinical sign for vitamin A deficiency (19)] during pregnancy was 2.9 times higher in urban poor women compared to rest of the urban population. This points towards the clear need to mandate frontline nutrition and health programme workers to provide vitamin A in liquid form, capsules or tablets to women who report night blindness. Additionally, mothers should be counselled to consume vitamin A-rich foods locally available in the community, such as (pumpkin, carrot, sweet potato, dark-green leafy vegetables, ripe mango, papaya, and jackfruit).

Iodine deficiency during pregnancy can impair motor, physical and mental development of the foetus and increase the risk of miscarriage (20). These impairments can be prevented if daily intake of adequately-iodized salt containing 15 parts per million (ppm) or more is ensured since food itself is a poor source of iodine. The present study showed that one-fifth of population belonging to the poorest urban quartile was consuming salt with no iodine, which was 2.8 times higher compared to consumption of non-iodized salt among rest of the urban population (8.7%). Hence, national and state-level Iodine Deficiency Disorders (IDD) Control Cells (21) need to strengthen periodic monitoring of the production and market availability of adequately-iodized salt. Regular community-based awareness-raising activities on benefits of iodized salt can be conducted through grassroots-level workers, non-government organizations, self-help groups formed under various schemes and through schools. To ensure adequately-iodized salt consumption at critical periods of pregnancy, the state of Gujarat provides adequately-iodized and packaged salt free of charge to all pregnant women during monthly village health and nutrition days (22). Packed and adequate iodized salt is made available at subsidized price through Public Distribution System in selected states of India, such as Chhattisgarh, Tamil Nadu, Gujarat, Rajasthan, Andhra Pradesh, Himachal Pradesh, Arunachal Pradesh, Sikkim, Tripura, and Karnataka (23). Scaling up such initiatives in other states will increase the access to adequately-iodized salt for urban poor populations.

### Conclusions

This paper highlights an utmost need for the use of disaggregated urban data and addressing nutrition inequities among women in urban India, which go unnoticed owing to the use of urban aggregate data at all levels. Given the high levels of undernutrition in women in the poorest urban quartile, it is suggested that the following may be done: (i) routine screening of women who are undernourished or suffer from three key micronutrient deficiencies, using field-based methods and instituting corrective measures; (ii) improving access to food subsidy through Public Distribution System (PDS) via better access to card-holders living below poverty line (BPL) or alternative approaches that are being currently debated or piloted; (iii) free distribution of iodized salt to pregnant women during health and nutrition days and/or universalizing its subsidy through PDS; (iv) institutionalizing nutrition and health counselling for mothers, using approaches involving fixed day, time, and venue and monitoring their coverage and quality for promotion of services and consumption of micronutrient-rich foods; and (v) resetting strategies for improving compliance to iron folic acid tablets and testing efficacy of strategies for alternative micronutrient food-based supplementation, including the strengthening of measures to prevent intestinal infections.

## ACKNOWLEDGEMENTS

The authors are grateful to Dr. S. Kaushik and Dr. Paromita Bannerjee for carrying out the analysis as members of the Urban Health Resource Centre team. The authors are also grateful to Dr. Fred Arnold and Dr. Sunita Kishor, senior researchers from ORC Macro, Dr. Kamla Gupta from International Institute of Population Studies, Mumbai, and Dr Massee Bateman for their input on methodology for analysis of urban subset of NFHS-3 data. The statistical analysis used in this paper was carried out by Urban Health Resource Centre with financial support through a grant from the United States Agency for International Development (USAID), India, through World Learning, USA, vide grant no. GSM 009 and subsequently through EMG-MCH Star under grant no. 4390-CR-08-0001 which ended in October 2009. Subsequent research and work for preparation of the manuscript has been done by the authors without any financial support from any source.
